# Mucinous Carcinoma of Ovary in a 15-Year-Old Girl, A Rare Case Report and Literature Review

**DOI:** 10.5146/tjpath.2024.13496

**Published:** 2025-09-30

**Authors:** Hazwa Karathanathodi Hamza, Nausheen Yaqoob, Khadra Ahmed Galaal, Aref Zribi, Ibrahim Al-Haddabi

**Affiliations:** Department of Histopathology and Cytopathology, Sultan Qaboos Comprehensive Cancer Care Centre, Muscat, Oman; Department of Surgical Oncology, Sultan Qaboos Comprehensive Cancer Care Centre, Muscat, Oman; Department of Medical Oncology, Sultan Qaboos Comprehensive Cancer Care Centre, Muscat, Oman

**Keywords:** Ovarian epithelial carcinoma, Mucinous neoplasm, Adolescent, HER2 Gene, Case study

## Abstract

Ovarian epithelial tumors are common in adults, and the median patient age at presentation is 55 years. In children, epithelial tumors are rare and mostly benign. Mucinous cystadenocarcinoma is reported in only 11 cases less than 15 years old.

This report describes the case of a 15-year-old postmenarchal Omani girl with ovarian mucinous carcinoma. She was admitted with severe epigastric pain and abdomen distension. CT scan showed a huge cystic lesion arising from the left adnexa filling the entire abdominal and pelvic cavity. The patient underwent laparotomy with left ovarian cystectomy and omental biopsy which revealed a 35 x 30 cm left ovarian cyst filled with turbid straw color fluid. Histopathology was reported as mucinous carcinoma. The patient later underwent cytoreductive surgery with left salpingo-oophorectomy, omentectomy, appendicectomy, and lymph node dissection that were negative for malignancy or metastatic disease. During follow-up, she developed a lymphocele in the pelvic cavity that was drained. There were no other significant issues during follow-up, as well as no evidence of recurrence or metastasis.

Epithelial tumors of the ovary are rare in young girls, with malignant tumors being exceedingly rare. Fertility-sparing surgery is adopted over radical surgery in these patients, even though the recurrence rates with this treatment protocol are high. All cases should be under follow-up to look for recurrence and timely management.

## INTRODUCTION

Mucinous carcinoma of the ovary (MOC) is an invasive mucinous neoplasm that accounts for 3-4% of all primary ovarian carcinomas. The median patient age at presentation is 55 years (1). Epithelial ovarian tumors (EOC) are rare in children, comprising 15% to 20% of cases (1). Mucinous ovarian tumors are even rarer in children, and most of the cases are benign/borderline tumors with mucinous carcinoma reported only in a few cases (2,3). Li et al reported a 14-year-old girl with mucinous cystadenocarcinoma with a literature review of 8 cases (4). In addition, 2 other studies have reported mucinous carcinoma with age less than 15 years (5,6). To our knowledge, this is the 12th case of ovarian mucinous carcinoma reported worldwide.

Multiple molecular alterations have been identified with copy-number loss of CDKN2A (76%) and KRAS mutations (64%) being the most common mutations (7). TP53 mutations occur in 64% of these tumors, a higher frequency than in mucinous borderline tumors; this implicates TP53 mutation in the progression from mucinous borderline tumor to mucinous carcinoma (8). ERBB2 (HER2) amplifications are detected in 18-26% of tumors and are known to co-occur along with the TP53 mutation (9). Furthermore, ERBB2 expression was found only in adenocarcinoma and was not detected in benign tumors (10).

Microscopic examination usually shows a continuum of architectural and cytological atypia, including benign, borderline, and carcinomatous areas (2). Carcinomas are characterized by two different patterns of invasion: expansile and infiltrative, each measuring at least 5 mm in linear extent (11). The expansile pattern is more common. The coexistence of both patterns has been reported. Carcinomas with expansile/confluent invasion have a better prognosis than those displaying infiltrative/destructive stromal invasion (11).

On immunohistochemistry, mucinous carcinomas are diffusely positive for CK7 in 90% of the cases, and variably positive for CK20, CEA, and CDX2. PAX8 positivity is usually focal and weak (12). p53 may show wild type or mutated staining patterns (8). p16 is generally negative or patchy positive (non-block-type immunoreactivity) (13).

We present the case of a 15-year-old girl who presented with abdominal pain and distention.

## CASE REPORT

A 15-year-old schoolgirl was referred from a private hospital with a history of severe pain in the epigastrium due to an overdistended abdomen. CT scan showed a huge cystic lesion most likely arising from the left adnexa and measuring 34 x 25 x 17 cm filling the entire abdominal and pelvic cavity. The cyst showed multiple septations and few enhancing papillary projections (less than 1 cm in size). The cyst filled the abdomen causing a mass effect. She underwent laparotomy and left ovarian cystectomy with an omental biopsy. Operative findings showed a left ovarian cyst filled with around 8.5 liters of turbid straw color fluid. Both tubes and the right ovary were normal.

Gross examination showed an opened cyst measuring 20.5 x 16.5 x 8 cm. The cut surface revealed multiple cystic locules of 6 cm to 17 cm and filled with clear contents. Papillary excrescences were identified. Extensive tissue sampling was performed.

**Figure 1:**
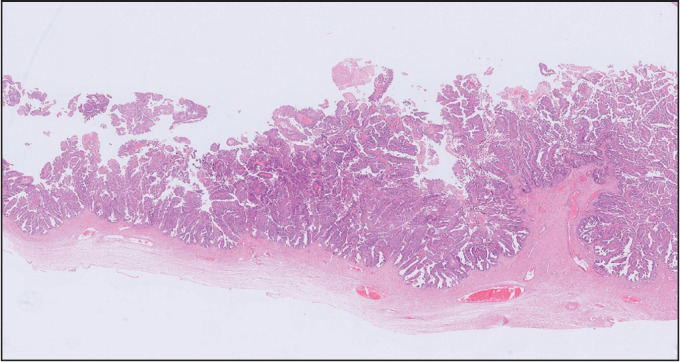
Complex branching architecture (H & E, 40X).

**Figure 2:**
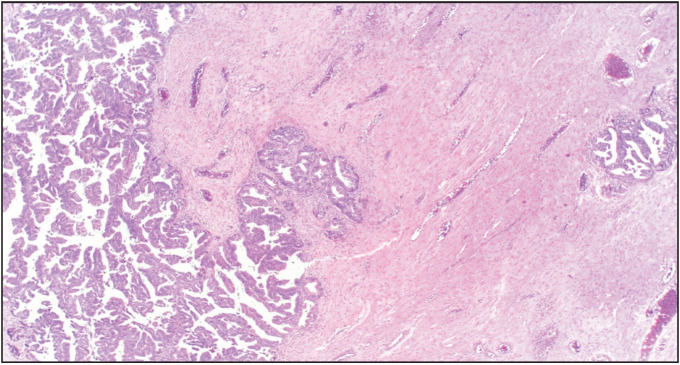
Focus of invasion (H & E, 100X).

**Figure 3:**
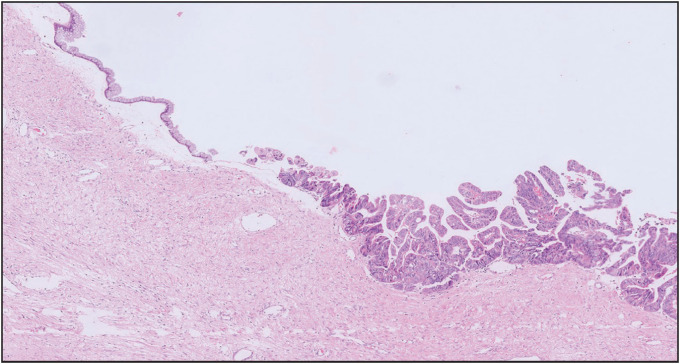
Transition from benign mucinous epithelium to borderline tumor (H & E, 40X).

Microscopic examination showed a neoplasm with solid and cystic areas exhibiting complex branching and crowded papillae with tufting and focal villous formations (Figure 1). The neoplastic cells exhibited moderate eosinophilic to vacuolated cytoplasm, irregular vesicular hyperchromatic nuclei with nuclear stratification, moderate to severe pleomorphism, and conspicuous nucleoli. Intraluminal necrosis was present. Frequent mitosis with atypical mitotic figures and necrosis amounting to intraepithelial carcinoma were identified. Infiltration of single cells into the papillary cores was seen. Focal stromal microinvasion which measured less than 5 mm in maximum microscopic dimension was identified (Figure 2). Adjacent areas showed a pushing type of invasion. In some areas, the cystic spaces were lined by tall columnar benign mucinous epithelium (Figure 3). Signet ring cells were not identified. Sex cord stromal, Brenner tumor, or germ cell components were not identified.

**Figure 4:**
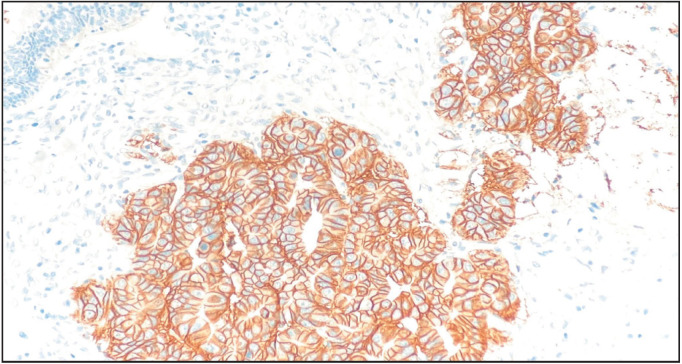
Tumor cells positive for HER2 (Score 3+).

Immunohistochemistry showed tumor cells to be positive for CK7, CK20, CDX2 & CEA. PAX-8 was focally positive. p53 showed strong and diffuse aberrant expression (mutant). Tumor cells were negative for p16, WT-1, Napsin-A, ER, PR, CA-125 and Vimentin. Ki67 showed an increased proliferation index of approximately 80%. HER2 by immunohistochemistry was Positive (Score 3+) (Figure 4). Immunohistochemistry (IHC) Testing for Mismatch Repair (MMR) showed intact nuclear expression for MLH1, MSH2, MSH6 and PMS2. The final diagnosis was reported as mucinous carcinoma with a background of mucinous borderline tumor and mucinous cystadenoma; pT1a.

The patient subsequently underwent further surgery with left salpingo-oophorectomy, omentectomy, appendectomy, and lymph node dissection being performed and was negative for malignancy or metastatic disease. The peritoneal fluid cytology sent during the procedure was negative for malignant cells. Her follow-up tumor markers were normal (CA125 - 22.83 KIU/L, CEA - 1.65, CA 19-9 - 34.01 KIU/L and Alpha fetoprotein - 0.824 KIU/L).

During follow-up, she developed a lymphocele in the pelvic cavity three weeks after the cytoreductive surgery and this was drained twice. The cytology showed abundant lymphocytes and was reported as negative for malignant cells. The patient is disease-free after 20 months of follow-up.

## DISCUSSION

Malignant and borderline ovarian tumors of epithelial origin are rarely encountered among prepubertal and adolescent girls (1–3). The frequency of malignant epithelial tumors is still rarer with only 14 cases reported in patients younger than 15 years (4).

Till date, only 11 cases of ovarian mucinous carcinoma have been reported in the literature in patients less than 15 years of age (Table 1) (1,4–7,14–17).

**Table 1:** Cases of mucinous carcinoma ovary in less than 15 years of age reported in literature

**Case no**	**Study**	**Age/Age range years**	**Pre/post-menarche**	**Operative treatment**	**Adjuvant therapy**	**Outcome**
**1**	Hernandez et al, 1982 (14)	10	Pre menarche	Left ovarian cystectomy	Melphalan	Disease-free after 22 months of follow-up
**2**	Gribbon et al, 1992 (15)	3 to 16	NA	Salpingo-oophorectomy	Pelvic radiotherapy	Disease-free 24 years after diagnosis
**3**	Skinner et al, 1993 (16)	2 to 16	NA	Salpingo-oophorectomy Omentectomy	Not given	Disease-free 6 years after diagnosis
**4**	Shankar et al, 2001 (7)	11	Pre menarche	Cytoreductive	Etoposide, carboplatin, bleomycin, cis-diamminedichloroplatinum and paclitaxel	Developed metastatic disease 6 months after recurrence and succumbed to illness after 2 years
**5**	Morowitz et al, 2003 (17)	9.9 to 17.9	Pre menarche	Surgery NA	Postoperative chemotherapy given	Died 1 year after diagnosis
**6**	Morowitz et al, 2003 (17)	9.9 to 17.9	Post menarche	Left salpingo- oophorectomy	Not given	Disease-free till last follow-up
**7**	Morowitz et al, 2003 (17)	9.9 to 17.9	Post menarche	Unilateral salpingo-oophorectomy	Not given	Disease-free till last follow-up
8	Gupta et al, 2017 (1)	14	Post menarche	Left salpingo-oophorectomy, omentectomy	Cisplatin and Paclitaxel	Disease-free till last follow-up
9	Drosdzol-Cop et al, 2022 (6)	14	Post menarche	Left salpingo- oophorectomy	Carboplatin and Paclitaxel	Disease-free till last follow-up
10	Alsereihi et al, 2022 (5)	11	Post menarche	Right salpingo-oophorectomy, omentectomy	Referred to oncology for further treatment	Disease-free till last follow-up
11	Li et al, 2023 (4)	14	Post menarche	Left salpingo-oophorectomy	Not given	Disease-free till last follow-up
12	Current study	15	Post menarche	Cytoreductive	Not given	Disease-free after 20 months of follow-up

Gribbon et al. studied 38 malignant ovarian tumors from age 3 to 16 years, of which one case was reported as mucinous cystadenocarcinoma, for 43 years (15). The patient underwent salpingo-oophorectomy followed by pelvic radiotherapy and remained disease-free 24 years after the diagnosis (Table 1). Skinner et al. reported a case series of 29 girls with malignant ovarian neoplasms of age 2 to 16 years (16). The study reported one case of mucinous cystadenocarcinoma presenting as Stage I disease (Table 1). Shankar et al. studied 3 cases of ovarian epithelial carcinoma in premenarchal girls, of which 1 case was reported as mucinous cystadenocarcinoma of the left ovary (Table 1) (7). Morowitz et al. evaluated the histopathology of 240 patients with ovarian masses for 14 years, and only three cases were found to have mucinous cystadenocarcinoma (Table 1) (17).

Various studies on mucinous carcinoma show most patients presenting with abdominal pain and distension (4,5), like our case presentation. Menstrual disturbances with vague symptoms have also been observed (4). The Li et al. study mentioned that 21% of patients with ovarian epithelial tumors were asymptomatic. This can lead to a late-stage diagnosis in many cases (4).

CA 125 has been widely used as a marker for epithelial ovarian tumors but its utility is debatable (1). The levels of CA125 are usually not elevated in Stage I disease (1). Furthermore, increased CA125 levels are observed in first-trimester pregnancy, endometriosis, pelvic inflammatory diseases, liver cirrhosis, pancreatitis, and non-ovarian malignancies (4,18). Hence tumor markers should be interpreted with caution and be correlated with radiology in the diagnosis of ovarian cancers (4,19). If CA125 levels are initially raised at the time of detection, they can be used as a marker for identifying residual or recurrent disease later at follow up (19,20).

The intra-operative examination of the uninvolved ovary, omentum, regional lymph nodes, and peritoneal wash cytology is important for staging the tumor (1). Though the adult staging protocols dictate mandatory lymph node dissections and biopsies of peritoneal surfaces, these procedures are often omitted in pediatric cases unless gross metastatic disease is present (17). Previous studies have shown the importance of prophylactic sampling of the uninvolved ovary during debulking surgery, given the significant incidence of bilateral disease (1,5,6). In the case presented by Alsereihi et al. the left ovary, fallopian tubes and omentum were normal, and the peritoneal fluid cytology was normal (5). Morowitz et al. presented a case of ovarian mucinous carcinoma with omental metastasis (17). In the present case, subsequent surgery with left salpingo-oophorectomy, omentectomy, appendectomy, and lymph node dissection was performed and was negative for malignancy or metastatic disease. The right ovary and fallopian tube were normal intraoperatively. However, a prophylactic biopsy of the right ovary was not performed in our case, considering the patient’s young age.

Fertility is of important concern in young patients. This reason substantiates the rationale of performing fertility-sparing surgery on these patients (1,4). The global recurrence rate for fertility-sparing surgery is 13% (21). Even though the recurrence rate was higher in fertility-sparing surgery as compared to aggressive radical surgery, these recurrences were amenable to salvage by subsequent surgeries (22). According to Aggarwal et al. low malignant recurrences have been reported more than ten years after initial surgery even in an adult patient population (23). However, Shankar et al. reported a case of ovarian mucinous carcinoma with recurrence and metastatic disease occurring 15 months and 21 months from initial surgery respectively (7). Therefore, like adults, young patients should also be kept under close follow-up to monitor recurrence and to treat with another salvage surgery (1).

The histopathology of mucinous tumors varies from benign cystadenomas to borderline tumors and cystadenocarcinomas. Mucinous cystadenomas account for 10-15% of all cases (4). Borderline tumors, or tumors of low malignant potential (LMP tumors), comprise up to 67 % of mucinous neoplasms (4). As in the current case, most of the tumor shows borderline features. In addition, our case also showed the borderline tumor to be arising in the background of a mucinous cystadenoma. Since the focus of invasive carcinoma is more than 5 mm, a final diagnosis of mucinous carcinoma was considered.

The available case reports on ovarian mucinous carcinoma have not performed immunohistochemical studies on the primary tumor (1,4,5,7,14,17). The present study showed an immunohistochemical profile in concordance with the reported literature (12) and like adult mucinous carcinomas evaluated in our laboratory. ERBB/HER2 expression is observed in the present case. Neil et al. have studied the case of a 29-year-old woman with recurrent ovarian mucinous carcinoma previously treated with capecitabine and oxaliplatin, which showed ERBB/HER2 amplification. After a second cytoreductive surgery, the patient was given Trastuzumab along with Carboplatin and Paclitaxel, followed by 1-year maintenance treatment with Trastuzumab. The patient remains disease-free after 3 years. This study supports the routine assessment of HER2 and highlights the potential of HER2-targeted therapy in combination with standard chemotherapy regimens in such cases (9).

Mucinous ovarian carcinoma (MOC) has a different natural history, molecular profile, chemosensitivity, and prognosis compared to other epithelial ovarian cancer (EOC) subtypes (24). The prevalence of MOC is very low, the same regimen used in all EOCs which is a combination of carboplatin, platinum‐based chemotherapy, and paclitaxel was used in this rare entity (25). Early-stage MOC has an excellent 5-year survival of almost 90%, while advanced MOC has a poorer prognosis and is less responsive to chemotherapy than EOCs, with 5-year survival rates of around 12% (26–28). Treatment of MOCs is only with surgical therapy for early stages whereas adjuvant treatment is required in late-stage carcinomas (29). Though stage I MOC has an excellent prognosis, it is believed that childhood tumors are far more aggressive than their adult counterparts and progress to advanced disease despite treatment (11,30).

In the present study, our patient was staged as FIGO stage IA and further adjuvant therapy was not recommended. The last imaging showed no evidence of any mass or collection in the pelvic cavity and no evidence of any distant metastatic disease. She is planned for long-term follow-up with imaging and tumor markers.

## CONCLUSION

Epithelial tumors of the ovary are rare in young girls. Most of the ovarian tumors in this age group are benign, with malignant tumors being exceedingly rare. Most patients have vague symptoms that are ignored and present mostly with metastatic disease. However, our patient had obvious symptoms of 3 to 4 months duration. Raised levels of CA125 with correlation to radiology findings will suggest the possibility of an epithelial ovarian tumor. Fertility-sparing surgery is adopted over radical surgery in these patients, even though the global recurrence rate with this treatment protocol is 13% (1,2). All cases should be under follow-up to look for recurrence and timely management.

No AI-assisted tools were used in the preparation of this work by any of the authors.

## Conflict of Interest

The authors declare that they have no competing interests.

## Funding

No funding was received for this work.

## Ethics Approval

We hereby state that an informed consent authorizing data publication was taken from the patient. The manuscript has been cleared by the institutional Ethics Committee (IRB&EC Project ID: CCCRC-84-2023).

## Patient Consent for Publication

Written informed consent was obtained from the patient and her parents for publication of this report and any accompanying images.
